# Autologous mesenchymal stromal cells embedded with Tissucol Duo^®^ for prevention of air leak after anatomical lung resection: results of a prospective phase I/II clinical trial with long-term follow-up

**DOI:** 10.1186/s13287-023-03545-8

**Published:** 2023-10-31

**Authors:** Marcelo F. Jiménez, María Teresa Gómez-Hernández, Eva M. Villarón, Miriam López-Parra, Fermín Sánchez-Guijo

**Affiliations:** 1 Service of Thoracic Surgery, Salamanca University Hospital, 37007 Salamanca, Spain; 2 Cell Therapy Area & Hematology Department, Salamanca University Hospital, Salamanca, Spain; 3 Salamanca Institute of Biomedical Research (IBSAL), Salamanca, Spain; 4 University of Salamanca, Salamanca, Spain; 5 Network Centre for Regenerative Medicine and Cellular Therapy of Castilla y León, Salamanca, Spain

**Keywords:** Mesenchymal stem cells, Mesenchymal stromal cells, MSCs, Air leak, Lung resection, Pulmonary resection, Lobectomy, Cellular therapy, Advanced therapies medical products: ATMPs, Tissue Col

## Abstract

**Background:**

Prolonged air leak (PAL) is the most frequent complication after pulmonary resection. Several measures have been described to prevent the occurrence of PAL in high-risk patients, however, the potential role of mesenchymal stem cells (MSCs) applied in the parenchymal suture line to prevent postoperative air leak in this setting has not been fully addressed.

**Objective:**

To analyse the feasibility, safety and potential clinical efficacy of the implantation of autologous MSCs embedded in Tissucol Duo^®^ as a prophylactic alternative to prevent postoperative prolonged air leak after pulmonary resection in high-risk patients.

**Study design:**

Phase I/II single-arm prospective clinical trial.

**Methods:**

Six patients with high risk of PAL undergoing elective pulmonary resection were included. Autologous bone marrow-derived MSCs were expanded at our Good Manufacturing Practice (GMP) Facility and implanted (embedded in a Tissucol Duo^®^ carrier) in the parenchymal suture line during pulmonary resection surgery. Patients were monitored in the early postoperative period and evaluated for possible complications or adverse reactions. In addition, all patients were followed-up to 5 years for clinical outcomes.

**Results:**

The median age of patients included was 66 years (range: 55–70 years), and male/female ratio was 5/1. Autologous MSCs were expanded in five cases, in one case MSCs expansion was insufficient. There were no adverse effects related to cell implantation. Regarding efficacy, median air leak duration was 0 days (range: 0–2 days). The incidence of PAL was nil. Radiologically, only one patient presented pneumothorax in the chest X-ray at discharge. No adverse effects related to the procedure were recorded during the follow-up.

**Conclusions:**

The use of autologous MSCs for prevention of PAL in patients with high risk of PAL is feasible, safe and potentially effective.

*Trial registration* No. EudraCT: 2013-000535-27. Clinicaltrials.gov idenfier: NCT02045745.

## Background

A postoperative air leak is defined by air scaping from the lung parenchyma to the pleural space after any kind of chest surgery. Although the incidence of any kind of air leak has been reported in around half of lung resection patients, the majority of these leaks ceases within few hours or days and air leak rates decrease to 5–20% by postoperative day 5 (excluding lung volume reduction surgery) [1, 2].

Air leaks that persist for longer than 5 days are reported and collected in the European Society of Thoracic Surgeons (ESTS) Database and in the Society of Thoracic Surgery (STS) Database, and this 5-day duration is commonly considered as the threshold for the definition of a prolonged air leak (PAL) after thoracic surgery. According to the last data reported in the ESTS Database Silver Book, the incidence of PAL following lobectomy is 9.4%, reaching 22.5% in lung volume reduction surgery [3]. In Spain, the results of a recently published study of the nationwide prospective multicentre registry from the Spanish Group of Video-Assisted Thoracic Surgery (GEVATS), reported an incidence of PAL of 11.86% after anatomical lung resection [4].

PALs are a burden to the healthcare system due to the associated increase in patient length of stay (LOS) and the cost of that LOS and of various necessary interventions [5]. In this regard, the incidence of severe PAL, defined as those that require an intervention in order to heal, has been noted to be around 5% after pulmonary resection [6]. Furthermore, its occurrence has been associated with a higher risk of pleural infections [7], readmissions [8] and impaired patient quality of life [5].

Several studies have identified risk factors for PAL [9–13], which include older age, lower body mass index (BMI), presence of pleural adhesions, surgeon’s experience, lower forced expiratory volume in the first second (FEV1), lower diffusion capacity for carbon monoxide (DLCO), steroid use and higher early postoperative air leak flow (mL/min) as measured on a digital chest drainage system [12–14]. Additionally, several PAL scores based on preoperative risk factors have been developed with the aim of identifying high-risk patients [15–18] who could benefit from earlier intervention.

As a result of the existing evidence on the deleterious consequences of PAL and the identification of risk factors, several measures have been described to prevent postoperative air leak following lung surgery. These measures include both surgical techniques such as the “pleural tent” [19] or the “fissure-less technique” [20] and the use of supports for parenchymal suture [21] or the use of tissue sealants [22–24]. Regarding tissue sealants, there are certain controversies. On one hand, there is evidence in favour of their use in the previously cited clinical trials, but a systematic review [25] advised against its routine use due to lack of conclusive proof effectivity and its high cost. On the other hand, interest in cell therapy has grown in recent years due to the ability of MSCs to differentiate into various cell lineages and act as anti-inflammatory and immunomodulatory agents, turning this cell type into a promising therapeutic tool with potential applications in regenerative medicine and tissue engineering [26, 27]. MSCs can be obtained from various sources such as bone marrow (BM), abdominal fat or synovium, and they have been assessed clinically for the treatment of various diseases [28, 29], including pulmonary disorders [30]. MSCs can be carried through a biological delivery system, such as fibrin glue, which acts as a temporary matrix that favours cell–matrix interactions and allows local and paracrine functions of MSCs. Furthermore, cell therapy strategies using MSCs carried in fibrin glue have shown promising results in regenerating nerve injuries [31] and treating lesions in bone or cartilaginous tissues [32], however, no previous studies have proven its potential to regenerate lung lesions.

There are reports of preclinical data on the effects of human adipose-derived stem cells on regeneration of damaged visceral pleural mesothelial cells on animal models [33]. However, there are no prospective clinical trials evaluating the role of MSCs, expanded in a Good Manufacturing Practice (GMP) facility, and released according to the International Society for Cellular Therapy (ISCT) criteria, in prevention of air leak after pulmonary resection in humans. For this reason, a small prospective phase I/II trial with a maximum sample size was designed in agreement with the Spanish Medicines Agency (AEMPS), to test this advanced therapy medical product (ATMP) in humans. This was also a final part of a translational research line started by the in vitro characterization of the interactions of MSCs with a fibrin sealant, Tissucol Duo^®^ (Baxter AG, Vienna, Austria) and their preclinical evaluation in a murine model [34]. The trial was supported by a public grant from the National Health System for non-commercial clinical trial development (see Acknowledgements).

The aim of this work was to assess the feasibility and safety of the use of autologous BM-derived MSCs embedded in a Tissucol Duo^®^ carrier applied to the parenchymal suture line in patients with increased risk of PAL undergoing anatomic pulmonary resection.

## Methods

We performed an open, single-centre, prospective, single-arm phase I/II clinical trial (EC Code: CSM/FAP/2012–EudraCT: 2013-000535-27). The trial was reviewed and approved by the Ethics Committee at Salamanca University Hospital and the AEMPS (Institutional Review Board protocol number: 13/1005). All patients signed the approved informed consent form, and all the procedures where in accordance with the principles of the Declaration of Helsinki.

Additionally, perioperative and long-term outcomes of two additional patients treated with MSCs after lung resection under compassionate use, outside of the clinical trial but with the same inclusion and exclusion criteria (see next section), were evaluated.

### Study population

Before surgery, all the patients diagnosed with lung cancer or with a suspicious pulmonary nodule were studied through an extensive work-up, which included physical examination, haematological and biochemical tests, electrocardiogram, computed tomography scan (CT) of the chest as well as abdomen, positron emission tomography (PET) scan and bronchoscopy. Further investigations were performed only if deemed necessary according to clinical findings or abnormal laboratory results. Patients with a previous history of cardiovascular disease or any suspicious changes in the electrocardiogram were referred for assessment by a cardiologist. Pulmonary function tests were performed in all patients. For this study, we reviewed only forced expiratory volume in the first second in per cent values (FEV1%) according to the patient’s age, gender and height.

PAL risk was assessed preoperatively according to the PAL score proposed by Brunelli [15] in collaboration with our team in 2010. Risk factors included in the score were: age > 65 years (1 point), BMI < 25.5 kg/m^2^ (2 points), FEV1% < 80% (1.5 points) and pleural adhesions (1 point). According to the obtained score, PAL risk was rated in four grades: A (0 points), B (1 point), C (1.5–3 points) and D (> 3 points).

Inclusion criteria in the trial were capacity to consent, age between 18 and 70 years old, patients planned to undergo anatomical lung resection (excluding pneumonectomy) and PAL score grade C or D.

Exclusion criteria included insufficient fitness to tolerate the surgical intervention, clinical or anaesthetic contraindication for surgery (American Society of Anaesthesiologist (ASA) score IV or V), presence of severe non-controlled systemic disease, pregnancy, positive serology for hepatitis B virus (HBV), hepatitis C virus (HCV), human immunodeficiency virus (HIV) or syphilis and absence or revocation of informed consent (Table 1).

**Table 1:** Inclusion and exclusion criteria

Inclusion criteria	Exclusion criteria
Capacity to consent	Insufficient fitness to tolerate the surgical intervention
Age between 18 and 70 years old	Clinical or anaesthetic contraindication for surgery (American Society of Anaesthesiologist (ASA) score IV or V)
Patients planned to undergo anatomical lung resection (excluding pneumonectomy)	Presence of severe non-controlled systemic disease
PAL score grade C or D	Pregnancy
	Positive serology for hepatitis B virus (HBV), hepatitis C virus (HCV), human immunodeficiency virus (HIV) or syphilis
	Absence or revocation of informed consent

Study population also comprised a patient cohort treated with MSCs after lung resection under compassionate use, outside of the clinical trial but with the same criteria. This cohort consisted of a high-risk cases of PAL who required a lung volume reduction surgery due to pulmonary emphysema. All of them signed a specific informed consent.

Since the study was designed as a phase I/II trial, a sample size (safety run-in cohort) of 6 to 12 subjects was estimated.

### Cell production and preparation

Cell production was performed in the GMP Cell Production Unit of the University Hospital of Salamanca, as previously described [35]. The ATMP was produced in our GMP Facility according to the Investigational Medicinal Product Dossier (IMPD) code PEI-13-072 approved by the AEMPS for this clinical trial. Briefly, 50–100 mL from BM were obtained from each patient’s iliac crest under sedation in a sterile environment in the operating room and transferred to the GMP Facility. Then, mononuclear cells were obtained after density gradient centrifugation and seeded in culture flask with alpha-MEM with 5% of platelet lysate to isolate MSCs, that were grown in vitro until the following release criteria were met: > 0.5 × 10^6^ MSCs/kg of recipient’s body weight, with > 80% viability and ISCT definition criteria regarding phenotype and differentiation ability [36].

Cell administration was coordinated with the thoracic surgery team. The two deep frozen solutions (sealer protein solution and thrombin solution) comprising Tissucol Duo^®^ were defrosted 24 h prior to use. Each solution was presented in a separate preloaded chamber of one double-chamber syringe. MSCs were collected, resuspended in saline and sent to the operating room properly labelled and in the shortest possible time. After thawing and warming up the Tissucol Duo^®^ to 37 °C, cells were resuspended in the thrombin syringe immediately before administration. The two solutions of the preparation (sealer protein solution and thrombin solution with the MSCs) were mixed during application.

### Surgical procedure and cell administration

Perioperative management was uniform for all cases during the study period. Preoperative antibiotic regimen consisted of a single dose of cefuroxime 1500 mg that was repeated 6 h later if surgery continued. Anaesthesia procedures were indicated and performed or supervised by a senior cardiothoracic anaesthesiologist, and all cases were operated on by the same team of senior thoracic surgeons, either through video-assisted thoracic surgery, anterolateral or muscle-sparing posterolateral thoracotomy approach.

Following anatomical resection of vascular and bronchial structures, mechanical (stapler) lung parenchymal suturing was done in all cases, and specimen removal was performed using a bag. Air leakage was then assessed by a water submersion test under standard airway pressure of 15–20 cm H2O. Application of the final product (autologous MSCs embedded in Tissucol Duo^®^) into the parenchymal suture line was performed after verification of grade 0 (absent, no apparent leak) or 1 (mild, countable bubbles) air leak according to Macchiarini scale [37]. The administration of the product was done topically over the parenchymal suture, either directly with the applicator of the double-chamber syringe in cases approached by open thoracotomy or with a spray system in cases approached by video thoracoscopy. To avoid the formation of excess granulation tissue, and to ensure gradual absorption of the solidified mixed solution, only a thin layer of the product was applied. Each patient received a dose of 1–2 × 10^6^ cells/cm^2^ of parenchymal suture. This final product was the only substance used for air leak prevention.

In all patients, a 24F or 28F chest tube was left in the apex at the end of the procedure and connected to an Atrium^®^ Ocean^®^ Water Seal Chest Drain device. This device has a scale in the water seal, graded from 0 to 5, to visually quantify the air leak.

Extubation was performed in the operating theatre, and patients were transferred to the cardiothoracic ward after an average of 6 h in the recovery room.

### Post-operative management

Post-operative analgesia was based on an epidural or paravertebral catheter that was inserted preoperatively, with a continuous bupivacaine and fentanyl infusion during the first 2 or 3 days, as well as intravenous analgesia as needed. Afterwards, oral paracetamol and non-steroid anti-inflammatory drugs were administered. Nursing care was also homogeneous in all cases and included incentive spirometry. All the patients were included in our specific pre- and postoperative chest physiotherapy programme, described elsewhere [38].

### Evaluation of post-operative air leak

Twenty-four hours after the surgery, air leak through the chest tube was assessed by two observers when the patient was in the cardiothoracic surgery ward and after performing chest physiotherapy. The patient performed deep forced expirations while timed during 1 min. In every expiration, we recorded the air flow observed in the chest drain’s graded water seal. This manoeuvre and evaluation of air leak was repeated every post-operative day until chest tube removal.

In addition to air flow quantification, the occurrence of cardiorespiratory complications and the presence of pneumothorax in chest X-ray were recorded on every post-operative day.

### Post-discharge follow-up

Post-operative follow-up was continued until at least 24 months, including five visits (at months 1, 3, 6, 12 and 24) where clinical outcomes (symptoms or sings of complications) and radiological assessments were recorded. Radiological tests included a chest X-ray to rule out the presence of pneumothorax in the follow/up visits at months 1, 3, 12 and 24 and a CT was performed at 6 months follow-up visit to detect potential pathological findings.

Although the established initial follow-up of the trial was 24 months, we followed all patients for an additional 5 years to assess safety and efficacy in the long term.

## Results

Six patients were screened for inclusion into the trial at our hospital between 2014 and 2016, and all of them were finally included in the study, as well as two cases in which MSCs were applied after lung resection as compassionate use in 2018.

### Patients and cell product

As already mentioned, the final population consisted of a single experimental treatment group of 6 patients (5 males and 1 female) with a median age of 66 years (range 55–70 years). Median FEV1% was 69.4% (range: 58.6–79.9%). Median BMI was 22.97 kg/m^2^ (range: 18.75–25.45 kg/m^2^). In four cases, pleural adhesions were suspected preoperatively. PAL score was rated as graded C (1.5–3 points) in two cases and as D (> 3 points) in four cases. The anaesthetic risk on these patients as classified by the ASA score was ASA II in one patients and ASA III in five patients. The main diagnosis was non-small-cell lung cancer (NSCLC).

MSCs were applied as compassionate use in two emphysematous male patients aged 64 and 73 years old, respectively, with low BMI and FEV1%. In both cases, PAL score was rated as grade D. Surgical procedure in these two cases consisted of lung volume reduction surgery for bullous emphysema.

Patient details are summarized in Table 2.

**Table 2:** Patient’s characteristics

Case Number	Age	Gender	FEV1 (%)	BMI	Pleural adhesions	PAL Score (grade)	Bronchodilatation treatment	ASA score	Diagnosis	Histological type	Pathological stage
1	62	Male	66.9	22.26	Yes	2.5 (C)	Yes	III	NSCLC	Large-cell neuroendocrine carcinoma	IIB
2	70	Male	71.9	25.45	No	2.5 (C)	No	III	NSCLC	Adenocarcinoma	IA2
3	55	Female	79.9	17.90	No	3.5 (D)	Yes	II	NSCLC	Adenocarcinoma	IIA
4	69	Male	78.6	23.68	Yes	3.5 (D)	No	III	NSCLC	Adenocarcinoma	IA2
5	64	Male	58.6	18.75	Yes	4.5 (D)	No	III	NSCLC	Adenocarcinoma	IA3
6	68	Male	63.4	25.00	Yes	3.5 (D)	Yes	III	Pulmonary nodule	Hamartoma	–
7	64	Male	34.9	17.44	No	3.5 (D)	Yes	II	Pulmonary emphysema	Bullous emphysema	–
8	73	Male	42.5	17.48	No	4.5 (D)	Yes	II	Pulmonary emphysema	Bullous emphysema	–

*FEV1* Forced expiratory volume in the first second, *BMI* body mass index, *PAL* prolonged air leak, *ASA score* American Society of Anaesthesiologist score, *NSCLC* non-small-cell lung cancer

MSCs expansion was successful in five cases included in the clinical trial and in the two cases in whom MSCs were considered as compassionate use. In one case, MSCs expansion was insufficient despite adequate BM harvest from the patient’s iliac crest in two different attempts.

MSCs were locally applied after the pulmonary resection in seven cases and no complications regarding its application during surgery were detected. The main characteristics of the cellular product applied are summarized in Table 3.

**Table 3:** Main characteristics of cell preparation and the cellular product applied

Case number	Amount of bone marrow obtained (mL)	Complications during bone marrow harvest	Number of MSC obtained after expansion	Number of MSC applied in suture line	Surgical procedure	Surgical approach	Complications during treatment
1	90	No	60 million	60 million	Left upper lobectomy	Thoracotomy	No
2	90	No	104 million	60 million	Segmentectomy (Left S1-3)	Thoracotomy	No
3	82	No	142 million	36 million	Middle lobectomy	VATS	No
4	95	No	255 million	60 million	Right upper lobectomy	VATS	No
5	90	No	Insufficient expansion	Not applied	Segmentectomy (Left S1-3 and S6)	Thoracotomy	–
6	80	No	120 million	60 million	Left upper lobectomy	VATS	No
7	90	No	100 million	60 million	Right upper lobectomy + wedge middle lobe + wedge S6	VATS	No
8	90	No	45 million	35 million	Wedge right upper lobe + wedge middle lobe + mechanical and chemical pleurodesis	VATS	No

*VATS* video-assisted thoracic surgery

### Clinical and functional results

PAL incidence was nil and median air leak duration was 0 days (range 0–2 days) among patients included in the trial. Only one patient presented pneumothorax in the chest X-ray at discharge. None of the patients who received MSCs developed any post-operative cardiorespiratory complication. Median length of hospital stay was 6 days (range: 4–6 days). None of the patients were readmitted to the hospital due to surgical complications within 30 days after discharge, and 30-day mortality was zero.

Regarding patients in whom MSCs were applied as compassionate use, one patient developed PAL that lasted 32 days and resolved without subsequent surgical interventions. However, no additional complications were detected in either of the two patients.

The main post-operative outcomes are described in Table 4.

**Table 4:** Main post-operative outcomes

Case number	Air leak duration (days)	Air leak quantification (Day 1)	Air leak quantification (Day 2)	Air leak quantification (Day 3)	Air leak quantification (Day 33)	Pneumothorax in chest X-ray at discharge	Cardiorespiratory post-operative complications	Post-operative mortality	Length of hospital stay	Readmission	Adjuvant treatment
1	2	2/5	1/5	0/5	–	Yes	No	No	6	No	Yes (Chemotherapy)
2	0	0/5	–	–	–	No	No	No	6	No	No
3	0	0/5	–	–	–	No	No	No	4	No	Yes (Chemotherapy)
4	1	1/5	0/5	–	–	No	No	No	6	No	No
5	–	–	–	–	–	–	Pneumonia	No	6	No	No
6	0	0/5	–	–	–	No	No	No	5	No	No
7	2	1/5	1/5	0/5	–	No	No	No	6	No	No
8	32	1/5	1/5	1/5	0/5	Yes	PAL	No	22	No	No

*PAL* prolonged air leak

### Long-term follow-up

There were no signs or symptoms of complications in any of the patients during the follow-up. One patient developed post chemotherapy leukopenia in relation to the adjuvant treatment applied which improved after drug protocol modification. We did not detect a pneumothorax on X-ray or pathological findings on CT in any of the patients during follow-up at months 1, 3, 6, 12 or 24 follow-ups.

One patient died before finishing the initial follow-up (2 years) due to disease progression (brain metastasis), which was diagnosed 19 months after the surgery. The patient who did not receive the MSCs because of insufficient cell expansion died 14 months after surgery due to respiratory failure secondary to pneumonia. The remaining four patients were followed for more than six years, and no pathological outcomes were reported in the routine clinical and radiological exams.

Regarding patients treated with MSCs as compassionate use, they were alive and no treatment-related complications or pathological findings were detected after a follow-up longer than 50 months.

The main long-term follow-up outcomes are summarized in Table 5.

**Table 5:** Follow-up

Case Number	1 month follow-up		3 months follow-up		6 months follow-up		1-year follow-up		2-year follow-up		Follow-up duration (months)	Status	Cause of death
	Pneumothorax in chest Rx	Complications	Pneumothorax in chest Rx	Complications	Pathological findings CT	Complications	Pneumothorax in chest Rx	Complications	Pneumothorax in chest Rx	Complications			
1	No	No	No	No	No	No	No	No	–	–	22	Dead	Brain metastasis
2	No	No	No	No	No	No	No	No	No	No	90	Alive	–
3	No	No	No	Post chemotherapy leukopenia	No	No	No	No	No	No	88	Alive	–
4	No	No	No	No	No	No	No	No	No	No	85	Alive	–
5	–	–	–	–	–	–	–	–	–	–	14	Dead	Pneumonia
6	No	No	No	No	No	No	No	No	No	No	76	Alive	–
7	No	No	No	No	–	–	No	No	No	No	52	Alive	–
8	No	No	No	No	–	–	No	No	No	No	51	Alive	–

### Complications and side effects

No major complications or adverse side effects related to the procedure (including heterotopic ossification, infections or tumours attributable to MSCs administration) were detected after the initial 2 years follow-up nor at 5 years.

## Discussion

In the last decade, cell therapy and tissue engineering have gained great interest in many disease conditions where no effective and curative treatment options are available, and numerous research studies have explored the potential of cell therapy for the treatment of several entities. In the field of pulmonary disorders, cell therapy with MSCs offers a novel and promising option for various acute and chronic lung diseases due to its immunomodulatory capacity, tissue regeneration, bacterial clearance and proangiogenic and antifibrotic properties [39].

Since the first isolation by Friedenstein et al. [40] in 1970, MSCs have become the most common cell type explored in regenerative medicine clinical development programmes. In the last decades, numerous preclinical studies have demonstrated an improvement in disease-associated parameters after MSCs administration for the treatment of several lung disorders, including chronic obstructive pulmonary disease, acute respiratory distress syndrome and idiopathic fibrosis [41]. More recently, a double-blind phase I/II randomized clinical trial conducted by Lanzoni et al. [42] demonstrated the safety and potential benefit of umbilical cord MSCs for coronavirus disease (COVID-19) acute respiratory distress syndrome.

Regarding the application of MSCs in injured lungs, in 2005 Rojas et al. [43] transferred bone marrow–derived mesenchymal stem cell to bleomycin-injured lung in mice, and they found that bone marrow stem cells were important in the repair process and that transfer of mesenchymal stem cells protects against the injury. Transplanted mesenchymal stem cells localize to the injured lung and acquire lung parenchymal cell characteristics. Similarly, in our previously mentioned pilot study in mice [34], we found that human BM-derived MSCs delivered using a fibrin sealant system after a lung parenchyma injury have the ability to integrate into the damaged parenchyma expressing different morphological features along the healing process. Furthermore, Shigemura et al. [44, 45] reported that autologous transplantation of adipose tissue-derived stromal cells (ASCs) to remnant lungs after lung volume reduction surgery for emphysema in a rat model resulted in enhanced alveolar and vascular regeneration through inducing hepatocyte growth factor (HGF) expression selectively in injured lung tissues. Kim et al. [33] described the morphological effects of transplantation of human ASCs to the damaged visceral pleural in a rabbit model and they concluded that ASCs improve the regeneration of mesothelial cells and can contribute to the treatment and prevention of alveolar air leak. In addition, cell therapy strategies using MSCs carried in fibrin sealant have shown promising results in regenerative medicine [31, 32]. Fibrin sealant provides a temporary structure that favours angiogenesis, extracellular matrix deposition and cell–matrix interactions and maintains the local and paracrine functions of MSCs, leading to tissue regeneration.

However, to date, no phase I/II clinical trials using MSCs embedded with a fibrin sealant have been conducted to prevent PAL after lung surgery in humans. In the current study, we include patients with high risk of post-operative PAL according to Brunelli’s score, but they were also high-risk patients according to their pathological diagnosis and preoperative comorbidities. Therefore, 5/6 patients included in the trial had a diagnosis of malignancy, which increased PAL incidence compared with benign pathology (OR: 1.45), and the two patients in whom MSCs were applied under compassionate use had diagnosis of pulmonary emphysema, which is also significantly associated with increased PAL risk (OR 2.35) [46].

Main strengths of our study include that, firstly, it employs MSCs produced in a GMP facility and released according to the ISCT criteria and fulfilling the requirements of the corresponding regulatory agency (AEMPS) to test this ATM in humans. Secondly, our trial is a prospective study where autologous BM-derived MSCs were mixed with a commercially available polymeric sealant (Tissucol Duo^®^) and applied in parenchymal staple line, so that the final product was homogeneous, and the preparation and administration techniques were easily reproducible. Thirdly, safety and effectiveness of polymeric sealants in preventing alveolar air leaks and shortening the duration of air leaks have been demonstrated in a systematic review and meta-analysis of randomised controlled trial [47]. Lastly, the final follow-up has been extended for more than 7 seven years in some cases, and the long-term results regarding safety and tolerability are excellent.

Our study has some limitations. First, since the study was designed as a phase I/II trial, the number of patients included is low, although we consider that it is enough to prove the safety and feasibility of the procedure since no secondary adverse effects have been reported during the follow-up. Second, conclusions on efficacy of the procedure must be cautiously considered since the therapeutic effect of MSCs embedded in Tissucol Duo^®^ in preventing post-operative PAL has not been compared with other sealants or alternative treatments. However, the results are promising and may encourage the design of larger phase II/III trials. Moreover, different cell doses have not been tested and may have an impact in short term outcomes and therapy efficacy. So that, further prospective trials should be conducted to evaluate this aspect.

## Conclusions

We have demonstrated the first proof-of-concept, confirmed the feasibility and safety of the procedure and provided a long follow-up (more than 7 years for three patients), which further guarantees the achievement of the primary objectives of a phase I–II trial, where absence of secondary effects is the most important finding.

It is also relevant to note that the incidence of PAL was nil among patients receiving MSCs and that only one patient presented pneumothorax at discharge. These outcomes point to the effectiveness of the application of MSCs in the parenchymal suture line to prevent PAL in high-risk patients. Additionally, we did not detect any clinical signs or symptoms of complications, or pathological radiological findings associated with the treatment during follow-up.

In brief, the results of the current clinical trial have demonstrated that cell therapy with autologous MSCs in prevention of PAL in high-risk patients is safe and feasible. Although the number of cases included in the trial was low, the duration of follow-up exceeded 5 years and no adverse side effects related to cell administration were detected.

## Data Availability

The datasets generated and analysed during the current study are included in this published article and its supplementary information files. The datasets generated and analysed during the current study are available in the Database_MSC_PAL repository, https://drive.google.com/drive/folders/16h4i9xohkytmTDXFc9V0lLcp8RCZeNRu?usp=share_link.

## Abbreviations

PAL: Prolonged air leak
MSCs: Mesenchymal stem cells
GMP: Good Manufacturing Practice
ESTS: European Society of Thoracic Surgeons
STS: Society of Thoracic Surgery
GEVATS: Spanish Group of Video-Assisted Thoracic Surgery
LOS: Length of stay
BMI: Body mass index
FEV1: Forced expiratory volume in the first second
DLCO: Diffusion capacity for carbon monoxide
BM: Bone marrow
GMP: Good Manufacturing Practice
ISCT: International Society for Cellular Therapy
AEMPS: Spanish Medicines Agency
ATMP: Advanced therapy medical product
CT: Computed tomography scan
PET: Positron emission tomography
FEV1%: Forced expiratory volume in the first second in per cent values
ASA: American Society of Anaesthesiologist
HCB: Hepatitis B virus
HCV: Hepatitis C virus
HIV: Human immunodeficiency virus
IMPD: Investigational Medicinal Product Dossier
NSCLC: Non-small-cell lung cancer
COVID-19: Coronavirus disease
ASCs: Adipose tissue-derived stromal cells
HGF: Hepatocyte growth factor
